# A Study of Environmental Factors in Low Vision Rehabilitation

**DOI:** 10.3389/fresc.2022.829903

**Published:** 2022-06-16

**Authors:** Turid Borgestrand Øien

**Affiliations:** Department of the Built Environment, Aalborg University, Aalborg, Denmark

**Keywords:** physical environment factor, holistic approach, low vision rehabilitation, lighting assessment and intervention, interdisciplinary collaboration

## Abstract

Healthcare has the past decades shifted from a narrow medical perspective to a more holistic, biopsychosocial perspective. Disability understood as a contextual condition constituted by the relation of the individual to their social and physical context. The disability model of the International Classification of Functions (ICF) contextualizes activity, participation, body functions and structure by including environmental and personal factors. However, illustrated by the consideration of the environmental factors as a neutral dimension, the dynamic interrelation of the individual parts of the system is rather unchartered. In 2017–2019, a lighting assessment was developed and tested on 60 participants in low vision rehabilitation. An action research project accompanied the pilot study from 2018. Ethnographic participatory observations of the low vision consultants in 15 consultations, semi-structured interviews, and a document analysis of the project material of the pilot project has been analyzed using the theoretical framework of science and technology studies. Mapping the physical environment showed a range of factors from spatial organization to luminaires and light bulbs. Moreover, in relation to specific activities, relevant factors were identified and assessed, and in the intervention adjusted to relevant personal and social factors. Identifying overlapping personal, environmental, and professional spheres illustrates the complexity of practicing rehabilitation in people's everyday lives. Acknowledging and coordinating different versions of lighting enabled low vision consultants to work across these spheres relationally. ICF was embedded in the practice of low vision consultants as a frame of reference, however, implementing this framework occurred through an assemblage of tools from different fields. The focus on lighting as an active element in low vision rehabilitation demonstrated a way to work across the personal and environmental to reduce the gap that caused disability. In everyday life, the physical environment was pivotal in the person–environment relationship and in enabling or disabling the individual. However, the physical environment was also key to the rehabilitation process, facilitating the individual's learning and change processes and reconfiguring their understanding and use of the environment. Consequently, the physical environment was not a neutral background to the other factors but rather enabling the rehabilitation and recovery processes.

## Introduction

The theory and practice of rehabilitation as a field are continuously developing. The introduction of the International Classification of Functioning, Disabilities, and Health [ICF, (1)] in 2001 illustrated a shift from understanding health from a medical model perspective to a biopsychosocial model. “A holistic approach to patient care” (2) comprised a shift from understanding disability as a condition of the individual to a condition of the individual situated in a specific social and physical context, associated with new views and valuation of human nature. By placing the individual at the center, expertise has changed from being solely a professional and medical matter to rehabilitation being acknowledged as a collaborative process occurring within the lived life of the patient and bringing their perspectives and expertise into play. Rehabilitation is conducted as a joint problem-solving process in which identification, investigation, goal setting, implementation, and assessment focus on hope, coping, positive self-perception, and the individual's perception of a meaningful everyday life to support “the mastery, learning, and change processes that characterize the work of rehabilitation” (3). The individual's process is known in the field of psychology as recovery and supports the individual in becoming more self-reliant, returning to working life, or living with a disability or impairment. Departing from the belief that all people have the potential for change and development, the recovery-based approach does not focus on the medical understanding of the patient as a passively disabled individual but rather the universal aspect of facing and coping with the situation to change and develop (4). The notion of holism is present in the new paradigm of rehabilitation in several ways, including the understanding of a *whole body* where conditions are related to both body functions and structures (1); the individual being situated in a *social and physical context* as part of the contextual factors in the ICF (2); and that the *rehabilitation process* is holistic regarding goals, duration, and results (5).

However, the contextual factors have not been adequately conceptualized and have consequently been difficult to measure (6) and operationalize in practice (7). Furthermore, meaningful application of the environmental factor component has been elusive. Hence, the environmental factors of ICF include the “physical, social, and attitudinal” environment, the physical seems to be absent or less implemented in the classification system. It is not mentioned in the three levels of functioning and in the qualifiers, it is reduced to standardized environment: A test setting or an environment “with uniform impact,” or “with precisely defined parameters based on extensive scientific research.” A generalized approach introduced to “neutralize the varying impact of different environments on the ability of the individual.” The interaction between a health condition and an environment has in ICF been interpreted as the effect of one on the other, while the interactive joint effect cannot be predicted from the sum of the individual effects (6). The version of holism embedded in the classification of functions still largely represents a medical understanding, a monistic materialistic view “where a whole is described as the sum of its parts” (8). Quantitatively assessed, reduced, and biomedically described parts, illustrated by the ICF's description of psychology as “functions of the brain,” exclude the interrelation between the body and psychological functions. Seen in this manner, disability or disease are understood in relation to a concept of normality, which falls short when addressing more complex or highly individualized psychological phenomena as Alzheimer's, anxiety, or chronic pain syndrome. The hierarchical system of codes reduces the complexity of the dynamic relationship between the individual and the environment, consequently limiting the holistic biopsychosocial perspective. To grasp the human–environment interaction holistically, Solli and DaSilvia argue that we need to acknowledge multiple spheres of reality (ibid.). The health impact of the environment, by increasing the experience of health or decreasing the experience of disability, is embedded in tacit knowledge more or less hidden from the traditional ICF perception of knowledge, why acknowledging health not only as a product of human function but also human experience would offer “a more inclusive, comprehensive, and holistic environmental factors component” (7).

Disabling and enabling environments have been a focus of universal design (UD) for the past 25 years. Parallel to rehabilitation, UD evolved from accessible design with special solutions for special needs to focus on abilities and inclusion from the mid-1980s onward. UD embodies a relational understanding of disability as a complex interaction between the individual and the social, cultural, and physical environment (9). Embracing the human experience and condition of living with disability, the relational gap model conceptualizes a more comprehensive understanding of disability as “emerging from the interaction between individuals and their social-material environments” and integrates knowledge from the social sciences, medicine, and the humanities (10). The gap model focuses on the interaction between the individual's abilities and the environmental demands in a specific situation, where the gap between the two creates the disability. A gap is prevented or handled by strengthening the individual's abilities and/or changing the environmental demands.

By embracing the role of the physical environment in enabling people with disabilities, UD holds a transactional process perspective acknowledging the interaction of the individual and their environment, including the wide variety of individual capacities that change over time (11). The holistic approach in UD has included the diversity of human interactions with the environment “fostering a more holistic understanding of the built environment” (12). Universalism is understood as “what is held in common by people,” not as a normative body, but “a universal human ethic that is simultaneously responsive to the specific, situated, nature of human subjectivities” (13). UD is not limited to buildings but rather includes, according to the UN Convention on the Rights of Persons with Disabilities, products, environments, programs, and services usable by people of different ages and impairments (14).

Acknowledging the different fields of knowledge and the limits of each professional field, UD emphasizes interdisciplinary collaboration. Rehabilitation professionals and user representatives provide important knowledge of the human diversity informing UD (9) and UD can facilitate and support rehabilitation with designs that are flexible, equitable, adjustable, and intuitive to use (13).

Another aspect adding to the complexity of rehabilitative practices, beyond the holistic and interdisciplinary, is the interaction between theory and practice. This interdependent relationship has been reinforced over the past decades as the practice becomes more professionalized and research aims to be more practice-based. However, the double move toward more holistic and interdisciplinary practice and the persistent silos predominating in research constitutes a paradox of the evidence-based practice. Environmental factors and especially the physical environment remain unknown territory for many rehabilitation professionals. In a Danish textbook on rehabilitation (15), the physical environment is represented by the different types of environments (home, neighborhood, workplace, and local environment) and by underlining accessibility and the use of assistive technology. “Very specific environmental factors can be taken into account and tailored specifically to the individual citizen's situation” (ibid.); however, these factors or their role in the rehabilitation are not further explained. Consequently, scholars call for knowledge concerning the “meaningful involvement” of the patient's knowledge of their life and preferences, their environments, and the mutual relationship between people and their environments (3).

Nevertheless, how do these paradigmatic shifts look in practice and how are holistic and interactional frameworks understood and enacted? Moreover, is it possible to bridge the fields of rehabilitation and UD to justify a study of the role of the physical environment?

Within the changing landscape of rehabilitation, innovative approaches have been mobilized, however the valuable practice knowledge associated to these efforts is often omitted in scientific representation and dissemination. The tacit practice knowledge includes both the translation of generic guidelines to specific settings and situations and the translation from specific situational conditions or considerations into explicit knowledge (16, 17). Because these knowledge translations are not made explicit, gaps in and resources for creating evidence and barriers in applying the evidence in practice arise (18). The application of knowledge and how it is accumulated differs by time, place, and culture, and a given profession has its own “disciplinary perspectives” that affect the understanding of problems and their solutions (13). Randomized controlled trials, which work well for well-defined groups of patients undergoing a well-known and described treatment under highly controlled conditions, are seldom available and not the ideal research design to study rehabilitation practice, which involves more complex and dynamically changing conditions in the patient group, the treatment, and the conditions (19). To relate to the lived experience of their clients “...much more information (than the diagnosis) is needed to understand the world in which people with visual impairment inhabit” (20). Meta-analyses, technological analyses, practice guides, and databases would be more appropriate to operationalize the current knowledge of rehabilitation practices, and richer descriptions and explorations of treatments, subjects, and physical environments would further improve rehabilitation practices (18).

The WHO states that the ICF “acts as a catalyst for change management as educators start modeling a holistic approach to patient care” (2). However, how can we move beyond the “neutralized environment” to recognize and work with the enabling and disabling aspects of our environments? How can this knowledge be operationalized in rehabilitative practice?

### The Context of Low Vision Rehabilitation Practice in Denmark

Lighting has been a key element of low vision rehabilitation for decades and innovations in the field of low vision and lighting have encompassed new lighting technologies and supportive aids; however, lighting assessments have traditionally been based on specifications tied to specific diagnoses, much in line with the medical model. Moreover, lighting interventions have been conducted in clinical settings and involved visual assessments combined with adjustments of the overall lighting to find the best lighting level for an individual positioned at a specific distance from a vision chart. Home assessments and smaller lighting interventions have been conducted but they have been unstructured, and the effects have been largely unknown. The small number of research studies on home lighting assessments in low vision rehabilitation that have been disseminated in the scientific literature include near-task lighting (21), lighting prescriptions (22), interventions with improved lighting (23), or the performance of and preference for different lighting levels (24). These studies have been limited to specific variables such as specifically pre-selected lighting or lighting levels, activities, spaces, or diagnostics, and guided by expert knowledge. Consequently, qualitative research of a “multifaceted approach to lighting intervention” that explores the experience of lighting environments has been needed (21).

In Denmark, rehabilitation has been a political focus and included in the Danish Executive Order on Social Services Act since 1998, and in 2015, a revision stated that rehabilitative initiatives for citizens with impaired functioning should be “organized and performed in a holistic and interdisciplinary manner” (25). Responding to this call, the Center for special education (CSU), Slagelse, initiated a pilot project entitled “Better Light, Better Living” (BLBL) in 2017 to test and assess a methodology and approach for a systematic lighting assessment and intervention. From 2017–2019, BLBL was tested with 60 visually impaired participants in three stages: a baseline assessment of lighting and activities in the home, lighting intervention in the lab, and a follow-up visit or phone call (26).

From 2018 to 2022, an action research project has accompanied BLBL, framing the pilot project as a case for investigating low vision rehabilitation and, particularly, the tacit embodied and embedded knowledge within the practice. The research project has involved sub-studies exploring the different types of contextual knowledge, including embodied knowledge of the individual, knowledge embedded in participants' routines and their interactions with environments, and knowledge embedded in the practices of the low vision consultants (16). The situated knowledge was identified, translated, and coordinated throughout the three stages regarding lighting in specific situations or activities, “linking the individual impairment and visual function to the physical and social context” (27). The light formed a boundary object of the physical environment, an embodied experience, and a shared social parameter, relational in all three matters (16). Within the BLBL approach, lighting design and implementation covered a wide range of the physical environment, from the geographically determined dynamic seasonal changes of daylight (17) to different arrangements, luminaires, and light bulbs. Even though the physical environment has played a key role in each of these analyses, the research objectives have been related to a rehabilitation process, recovery process, or co-design process. What can we learn about the environmental factors from a case like this, and how can it support the holistic effort in rehabilitation?

## Theoretical Framework

Exploring the role of the environment in professional rehabilitation practice has, due to the interdisciplinary character of the action research project, been informed by fields beyond traditional health research. Drawing on the social constructivist approach of science and technology studies (STS), and actor-network theory (ANT), the human-environment interaction is emphasized as the core of these approaches' acknowledgment of socio-material interaction and actor-networks as wholes constituted by human and non-human actors (28). These studies do not aim to provide deterministic causal explanations of how science or technology influence society but rather to “make available resources for thinking systematically about processes of sense-making... reintegrated into explanatory projects that conform more accurately to the lived experience of modern societies” [(29), p. 38]. In this process, there “is no social order … [but] endless attempts at ordering” (30). Material artifacts, humans, and conventions are mutually interdependent in effecting and affecting one another. Unlike the reductionism of the medical approach, this could cause issues of the overwhelming proportions of holism (29). Therefore, mapping these actor-networks requires attention to the relevant actions taken and identifying and following the relevant actors, which allows the study of complex and dynamic relations. In her study of atherosclerosis and the different versions of this phenomenon enacted in different practices at a Dutch hospital, Annemarie Mol developed the notion of the “body multiple” (31). This approach acknowledges that different ontologies coexist across professional and personal practices as different versions that, in each of the different settings, are ignored, excluded, acknowledged, included, distributed, or coordinated. These enactments involve entanglements of methodologies, treatment paradigms, and knowledge, but also instruments, representations, blood, flesh, and, not least, how “people live with diseases” (ibid.).

## Materials and Methods

Ethnographic studies in healthcare can help us illuminate “the organizational and interactional processes through which health care is delivered” (32). BLBL has been studied as an ethnographic, mixed- methods, prospective, and longitudinal designed “in-depth, detailed, holistic case study” (33). The empirical material have included *fieldnotes and visual material* from participatory observation of 15 consultations (8 home visits and 7 lighting lab sessions) following one of the two low vision consultants, a visually impaired participant and his/hers accompanying family member in the home environment or in the facilities of the low vision services; *project documentation* from all 180 consultations (home visits, lighting lab sessions, and follow up with the 60 participants), and *transcriptions and fieldnotes* from a series of semi-structured interviews with the two low vision consultants. The 15 consultations have been considered as sufficient for investigating the role of the environmental factors and its dynamic interactions within the intervention. Sample size calculation or other defined robustness have not been considered.

Observing and identifying the practical knowledge of the professionals included their approach to the participants, their use of the schemes and technologies within the project framework, the involvement of the individual, and the social and physical context. Beyond the work of the low vision consultants, the observations involved the spaces, physical traces of the participants' use of the space, and adjustments or adaptions of spaces or arrangements. Observations also included the dynamics and interrelation of the users, the space, and any material artifacts relevant to the specific activity. As lighting is most often used in “tacit, normally unspoken about ways to make, maintain, and improvise atmospheres of home” (34), observations of the consultants and participants moving around the home environment focused on their interaction with and articulation of the lighting and the environment. The research focus on the role of the physical and social context in the intervention has been informed by architectural anthropology, recognizing the home environment as “part of wider socially situated practices [that] helps us understand the shifting dynamics at play” and located between “the technical, social, and individual, insisting on a holistic understanding” (35). The initial findings of the project have been addressed and discussed in a series of qualitative semi-structured interviews with the two low vision consultants of 1–2 h discussing outcome, measures, intervention design, tacit knowledge, and dissemination. The interviews have been recorded and transcribed.

**Figure 1:**
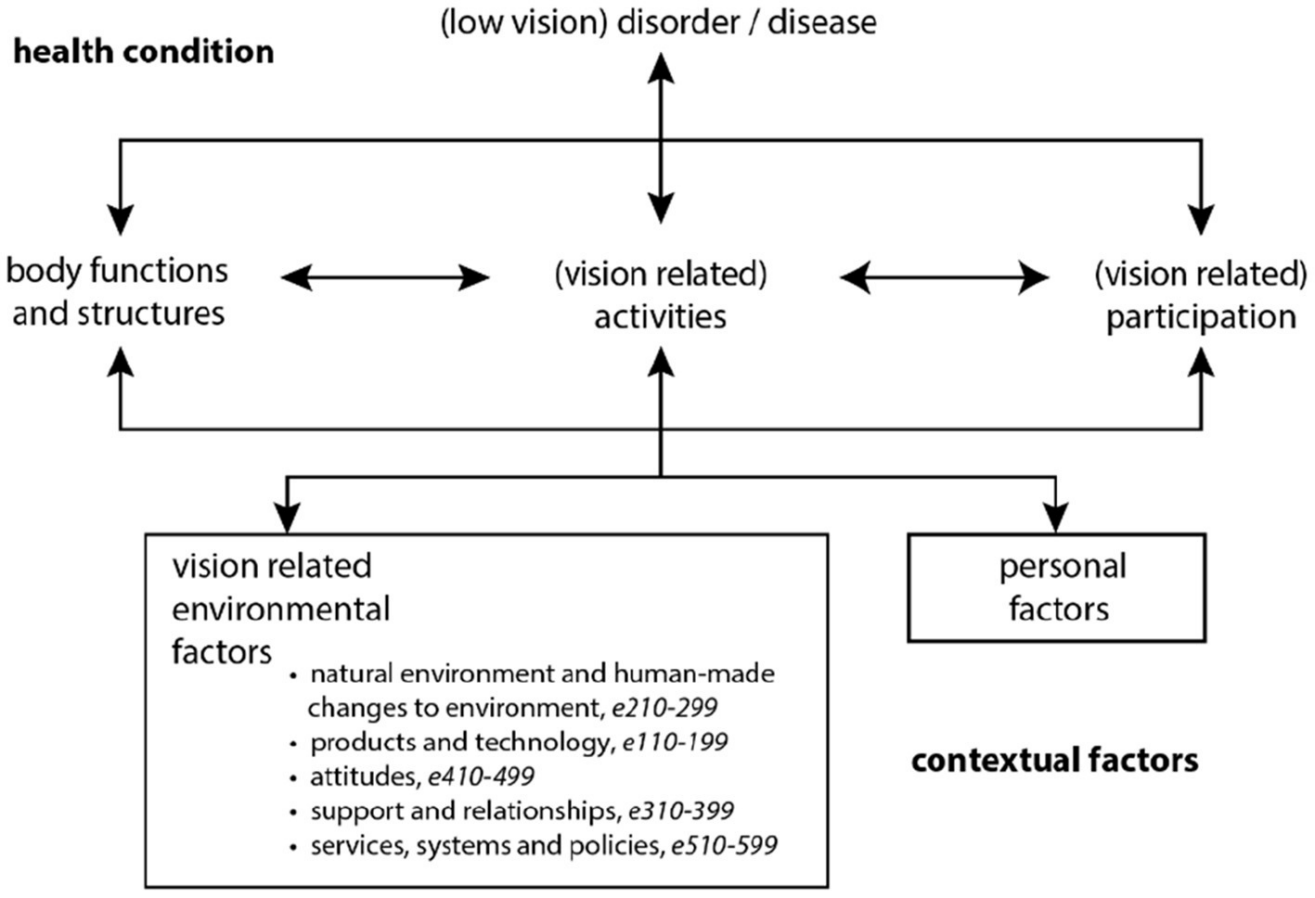
The ICF disability model [(1), p. 9] adapted for low vision rehabilitation, including the classification's further subdivision of environmental factors.

Thorough descriptions have been drafted from the observations based on field notes, transcriptions, and visual materials. An analytically focused sampling was conducted to identify and describe (a) the active environmental parameters of the intervention and (b) patterns and themes of the interactions and change processes. Finally, a theory-focused concept sampling was conducted to analyze and discuss the relation and fit of these results and the framework of ICF, depicted in Figure 1.

The BLBL pilot study was conducted as a quasi-experiment without a control group, where all participants received similar and non-invasive interventions. The comparison of baseline and endline measures was included as a quality assurance of the intervention, and consequently it did not require review, approval, or permissions from Ethics Committees or Institutional Boards. For storage and use of personal information the trial was conducted according to Danish legislation and adhered to requirements of the data monitoring committee. Informed consent was collected from the 60 participants as part of the overall BLBL framework, the participatory observation was announced to and approved by the participants before our visits, and the focus of the observations on the work of the low vision consultants and the overall BLBL project was repeated when we arrived. Personal information of the participants has not been used in the ethnographic research study. Moreover, the framework for the research collaboration with CSU was defined in a legally binding cooperation agreement between our institutions, where dissemination of research results has been shared and reviewed within the project group before disclosure.

As an outsider to the health profession, I lack the professional understanding of how health is practiced and privileged focus on the process of recovery or rehabilitation. Conversely, as an architectural researcher equipped with anthropological and socio-constructivist frameworks, the attention has been paid to the role of the physical context, however entangled and situated in the everyday lives of the participants and the professional practice of the low vision consultants: As a way for my position to contextualize the ‘usual suspect’, the built environment, in social and professional overlapping spheres. One of the gifts of qualitative empirical studies, as a case study or an innovative project, is that it can enable us to recognize the different spheres, and how they interact and affect one another. Making the know-how from the “other” discipline explicit, here discussing the ICF framework in a metatheoretical matter within a rehabilitation journal, is also perhaps an outsider-position, however inviting for a joint exploration of the field of rehabilitation.

## Findings

Where the overall objective of the low vision consultants in BLBL was to improve the participants' quality of life, the interest in this paper has been to identify interactions within the processes and, more specifically, the role of the environmental factors.

### Identifying Contextual Factors and Actors of the Physical Environment

Identifying the relevant environmental factors to BLBL starts with the seasonal changes. Based on their prior practice knowledge, the consultants knew that the issues regarding lighting primarily arose in the dark winter months, and why the pilot study was conducted in this period of the year. Being located in the northern hemisphere, these seasonal conditions affect our everyday lives and several participants mentioned natural lighting and daylight as important for their functioning (17); many described individual preferences regarding natural lighting, whether daylight, overcast, sunlight, or twilight (27). Furthermore, at a *building scale*, the typology, structure, and orientation of the building as well as the dimensions, size, and position of windows in the façade influenced the amount of daylight present. Additionally, obstacles outside the building, the floor number in a multi-story building, and the spatial distribution of the apartment affected the distribution and amount of daylight available. The overall composition, dimensions, proportions, and organization of *spaces* affected each specific space. Ceiling height and depth affect the experience or atmosphere of a space, creating different characteristics (small, large, intimate, formal, etc.), while interconnected spaces, thresholds and openings, and the fixed interior provide coherence or contrast. At the next level, were material artifacts such as furniture of different shapes, materials, and colors and the amount and position of furniture, leaving more or less free floor space to move in. Some living rooms were equipped with sofa arrangements in dark leather, combined with long, dark curtains, while other living rooms were sparsely furnished and kept in pale colors. Different wall paints, wallpapers, panels, different finishes, and amounts of pictures and other artifacts on the walls affected how light was reflected from the surfaces. Likewise, the color and finish of the floor and ceiling, curtains, and other types of stationary or flexible shading, overhang, awning, film, or blinds also affected the environmental context.

The main lighting parameters can be divided into *light space, luminaire*, and *light bulb*. As this was the focal point of BLBL, the aspects within these categories were thoroughly explored and mediated throughout the stages of the program. *Light space* can be described as the illuminated space established in a darker surrounding: the overall space conditioned by size, form/distribution, and orientation/direction, comprised of daylight or artificial light, direct or indirect light, and its interplay with shadow. Different light spaces can be arranged to integrate or create isolated isles of light and can be coordinating or contrasting if there is a large difference in luminance. The *luminaire*, or what we know colloquially as a lamp or lighting device, includes the electrical device, the fixture that holds a light bulb, diode, or tube, and often a system of shades, screens, and/or diffusers. A range of different shapes, sizes, and proportions were represented in the homes, differences that influenced how the light was emitted and illuminated the close surroundings and surfaces. Consequently, the position and orientation of a luminaire affected the light space, the material of the shade or screen was another important variable of how the light was distributed. A translucent material, such as an opaque or frosted glass lamp, spread the light and made the lamp appear as a luminous object, while a metal shade concentrated the light and directed it and provided more light on the surface of, e.g., a table. Luminaires was represented in many different typologies, styles, and designs from industrial, ceiling-mounted light panels to traditional designs or aesthetic, neatly geometric or organic and sculptural lamps. These was either mounted on the ceiling or wall, standing on a table or on the floor, was either fixed, flexible, or mobile and could be switched on and off by switches on the lamp or wall or by remote control. Finally, the *light bulb* (or diode and tube) was pivotal for the light space, especially the relation of the light source's intensity to the overall luminaire and its close surroundings. Also, the bulbs came in different shapes, size, and lighting technologies, dimmable or fixed, and of different intensity, color temperature, and rendering.

**Figure 2:**
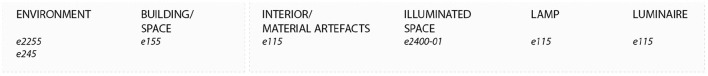
Mapping of the physical parameters and its corresponding classification codes in ICF.

Two spatial categories have been added to the ICF framework for the environmental factors, building/space and illuminated space. Building/space as an intermediate between the environmental (daylight, location, and orientation), and the products and technology of daily use, including interior/ material artifacts. And illuminated space as subcategory to building/space, as an intermediate between the luminaire, light source, and the overall physical setting. As seen in Figure 2, these physical parameters have corresponding ICF classification codes: seasonal changes (e2255; e245), building scale (e155), and the illuminated space (e2400-01). The interior, lamp, and luminaire share the same code (e115).

### The Professional Navigation and Coordination of the Environmental and Personal in the Rehabilitation Process

#### In the Home Environment

*When I enter a house, I look around. “Okay”… As soon as I enter, I scan the room for any glare, flicker, large contrasts—how does my eyes adjust. Moreover, the shape and design of the lamps, the light bulbs… This just happen automatically, no matter whether the light is good or bad…*

Typically, the consultant would have some impression and expectations of the specific case before the first visit. This would include prior knowledge from the participant's journal concerning their vision (a), including impairment as well as knowledge of the residential area, and brief knowledge of the situation from an initial phone conversation and a self-reported visual function questionnaire (VFQ39) (b). Furthermore, the subconscious scanning described above would often start before entering the home: Recognizing the building and typology of housing and the close surroundings would form initial impressions and input that constituted a baseline (c). The first impressions of the interior space provided further input of the specific participant's everyday life (d). Because, as stated by one of the consultants: “…then we explore the challenges of the visually impaired individual... because sometimes their issues are far from my initial personal guess.” The narrative interview focused the conversation on the personal (e), in which the participant was encouraged to share their experiences with activities where the current lighting condition and their visual impairment were misfit and disabled them. The personal could involve psychological, neurological, spiritual, cognitive, physical, and biological concerns as well as aspects regarding their vision that arose during the conversation such as light sensitivity, eye strain, contrast vision, or issues regarding adaption, color appearance, and the stability or flux of their condition. Furthermore, more practical issues regarding their use of optics and aids such as glasses, magnification, or distancing were discussed. Three activities were selected for further assessment, where the participants specified personal aspects such as handling self-care, preparing or consuming a meal, or hobbies and social aspects such as communicating or socializing (16) (f). Activities were situated in different specific parts of the home environment and concerned specific illuminated spaces. The participants rated their performance of each activity by importance, performance, and satisfaction on the Canadian Occupational Performance Measure (COPM), shifting the focus to motivation (g). The motivational aspects were both internal, related to the individual's hopes, dreams, and aspirations regarding the implications of potentially being able to resume to these activities in general or with less effort, and external, linked to specific social and physical activities. The actual relevance of the social context differed by case, as some family members were more actively involved than others; however, in general, the social context constituted an external motivation.

Subsequently, the assessment continued in the specific physical setting of each activity (h), enabling the participants to get more specific regarding the difficulties and workarounds for handling an activity. These situated conversations often involved narratives of attunements, culturally and socially informed aspects of settings such as wanting to create a specific atmosphere for social occasions like “hyggebelysning” [cozy lighting] for a family dinner or as part of an evening ritual (17). Situating the activity within the interior arrangements, material artifacts, illuminated space, and with the specific luminaires and light bulbs (i), the visual function was assessed by two measures representing a baseline.

With the participant positioned in their activities, the consultants proceeded with the lighting assessment. Often initiated with an implicit scan of the close surroundings: noticing the illuminated space, the distribution and direction of light, the relationship of natural and artificial light, and the relationship of direct light, indirect light, and shadows. The activity further enabled a situated experience of light, shared by the participant, the family member, and the low vision consultant. The walkthrough, demonstrations, and practical performances enabled reflections and discussions as frustrations or comments on their own feelings or performances were shared and discussed. Observing from the position and field of vision of the participant, the consultant could note whether glare, from reflections of the luminaire or a directly visible light source, was an issue, or observe the specific luminaire, its type, shape, material, dimensions, proportions, location, orientation, and mobility, in relation to the participant and the activity. The characteristics of the light source were also assessed, including the type, material, luminance, and relation to the overall luminaire, and again the relation to the participant and activity. Furthermore, the consultant could consider the lighting demands in relation to other activities in the same setting, whether it was appropriate across activities or there was a need for adjustable or different types of lighting. The relevant aspects of this implicit scanning were addressed and discussed with the participants during the assessment.

Beyond the subconscious scanning and positioning to detect potential issues in the home environment and possibilities for improvement in the overall and specific settings, different tools and measures were applied to assess the light (i). Luminance was measured on surfaces with a light-meter or the spectral distribution of wavelengths was measured with a spectrometer. These devices were also used during the dialogue to demonstrate, e.g., the features of an energy-saving compact fluorescent lamp (CFL) or an insufficiently illuminated dining table. The CFL's spectral distribution—illustrated by a range of peaks in the display of the spectrometer—was compared with the more even distribution of natural light or an incandescent bulb or LED. The illumination in a specific location was illustrated by comparing the lux measurement to a measurement in a setting with more sufficient lighting or to the general recommendations for lighting in work environments. The explicit measures of the lighting levels (lux) in the central positions of the settings were recorded on a provisory sketched plan showing the simple spatial layout. Along with photos taken of the different situations, this constituted the documentation of the home environment.

Often conducted as the last element of the first visit, the accompanying family was offered a guided simulation of the visually impaired person's visual experience (j). An application for simulating different diagnostics and conditions enabled the consultants to recreate visual acuity and impairment similar to the individual, and by adding filters to the settings sequentially they could specify and explain the different conditions. In a pair of VR/AR goggles, the family member had the visual condition explained in a virtual reality representing the lighting lab of CSU, followed by an exploration of the home environment in an augmented reality setting situating the visual condition to the specific home environment and lighting condition. Furthermore, the demonstration often inspired engaged conversations between the visually impaired person and the accompanying kin and, thereby, another processual loop linking the individual, social, and environmental.

**Figure 3:**
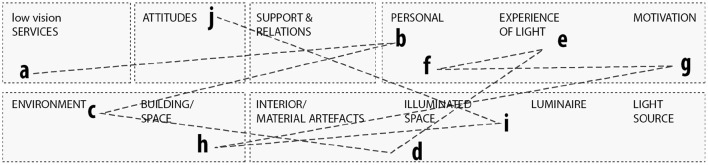
Mapping of the trajectory of the implicit and explicit sequences of the assessment in the home environment placed in relation to extended ICF categories.

Figure 3 shows the trajectory of the typical home visit, regarding the distribution and diversity of the relevant actors, that beyond the parameters of the physical environment involved personal and social parameters. In addition to the categories of the ICF classification, the spatial characteristics of the home found in the living spaces and the illuminated space, included both material artifacts (walls, surfaces, furniture) defining the space and human and non-human substances inhabiting it. In these first steps of the assessment, the interaction with the participants focused on the visually impaired person's overall experiences with and use of lighting in their everyday routines, relating this to both their social life in the household and their overall feelings about their impairment.

#### Lighting Lab and the Clinic

Moving on to the lighting lab, the lighting intervention was framed as a collaborative process between the low vision consultants and the participating visually impaired and his/her company. The lighting intervention was intended to recreate a lighting condition similar to the specific settings from the activities in the home environment. The consultant would have “prepared the stage” before the participants arrived and, with the visually impaired person positioned in the scenario (k), different lighting, lamps, and arrangements could be tested (l). If the low vision consultant considered a different luminaire as part of the solution, an alternative was compared to the original by demonstrating one and then the other (m) with the participant seated in the same position. This allowed the individual to pay attention to nuances of how the two conditions affected their visual perception and ability to perform the activity (n). In this testing phase, different relevant attributes (o) were involved such as food on a plate, crossword puzzles, magazines, or if the activities concerned using appliances or furniture such as the kitchen surface, washbasin, or wardrobe, these were incorporated into the practical testing. Some participants were encouraged to bring own relevant artifacts to the lighting lab. One woman, who had faced difficulties perceiving form and substance in her current lighting, brought samples of clay, as she wanted to test whether a different lighting could help her distinguish different material characteristics (p). Similarly, differences in lighting quality by color or intensity, were tested and compared by introducing different light sources (q). The line of tests was informed by the embodied experience of the participants, managed by the tacit knowledge of the consultant adjusting to the individual's interest and mental or physical energy (r).

Leaving the clinic and the lighting lab, the participants left with a specified lighting prescription for the suggested lighting adjustments for each activity, including printed photos from the home environment where the consultant had outlined the shape, position, and direction of the luminaires or outlined if any of the existing lighting should be moved or removed. These contextualized recommendations eased the involvement of support and relations (s) to implement the recommendations. Both by the participating family or friends, but also personal assistants, electricians, or lighting professionals.

**Figure 4:**
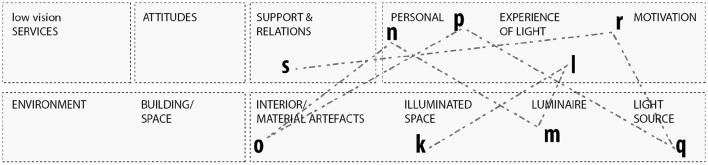
Mapping of the trajectory of the implicit and explicit sequences of the assessment in the lighting lab placed in relation to extended ICF categories.

The lines mapped in Figure 4 represent the sequences of the lighting intervention in the lighting lab. As a continuation of Figure 3, the trajectory show that the consultants continuously worked across personal and environmental issues, aligning them to the situated activity.

### Relational Understanding of Light

The empirical material of the processes in the BLBL program demonstrates a multifaceted and relational understanding of light. Where the assessment in the home environment facilitated the individual's reflections on their own current performance of daily activities, the intervention focused on the embodied knowledge and new or resumed abilities enabled by the adjusted lighting. Beyond physics, the lighting was related to the specific activity and experienced by the individual through their visual abilities and affected by their hopes and desires, and change could be enacted in the individual by adjusting the social and physical context.

However, in many cases, the changes occurred in all three aspects. It was close to impossible to say what started and what affected which aspect. In some cases, no changes were made to the light but rather to the way the light was used. For others, the family or kin accompanying the visually impaired person gained a better understanding of their issues and were motivated to implement the changes. The problems identified were related to specific activities and visual functions and supported different interpretations and uses of light (16).

The consultants have highlighted the importance of active involvement as one of the main findings of BLBL; rather than just telling people what to do, the assessment and one-on-one testing enabled the participants to take action and make changes:

*When we started the project, we were not at all aware of the extent of the education or coaching in BLBL. However, it is a great deal, for them to really understand it….I think that is one of the pivotal differences from our previous practice regarding lighting, that earlier we [as the experts] made the decisions for them, now we enable people to make their own decisions*.

## Discussion

### Enabling and Disabling Environments

Within the ICF classification, the “physical, social, and attitudinal” environmental factors are recorded and coded individually as facilitators or barriers to human functioning and their effects are assessed by the functioning of “the person overall, to each ICF component, or to performance and capacity” (2). Even though several of the parameters are described in the ICF classification codes, it is often related to isolated variables, as for e155 where design, construction and building products and technology is specified to “entering and exiting” (e1550), “gaining access” (e1551), “wayfinding” (e1552), or “physical safety” (e1553). However, the study expands the understanding of these factors beyond a neutralized environment and shows that these components are interlinked and dynamic: The physical environment has a huge effect on the social and the personal, and in the process of BLBL they are operationalized in relation to one another.

Whiteneck and Dijkers (6) suggest that to operationalize the environmental factors of the ICF, we should abandon the traditional tests and rather ask two questions: First, the level and type of functioning the individual desires and second, whether there are environmental barriers impeding them (nuanced by quality, quantity, or ease). This has more or less been the BLBL approach, embedded in the narrative interview, COPM, and lighting assessment and intervention. The interview was focused on lighting; however, this environmental parameter was operationalized and used as an active part of low vision rehabilitation to support the personal recovery process of the individual participant. In this manner, activity issues and the related lighting problems were aligned with the individual's perception of a meaningful everyday life and focused on the support of the mastery, learning, and change processes at stake (3). The environmental factors and the individual were considered active and dynamic aspects responsive to change (4) and lighting was a key actor in the rehabilitation process that was used to facilitate rehabilitation and change processes for the individual's recovery.

The focus on activity and usability throughout the problem-solving process made the environmental factor of lighting operationalizable, allowing for solutions that fit the principles of universal design (UD): *equitable, flexible, simple and intuitive use* with *low physical effort* within the required *size and space*, providing *perceptible information* and forecasting the potential *tolerance for error* (12).

### Holistic

Both the ICF and UD are models aiming for a more holistic approach to rehabilitation or design. The holistic approach in UD prioritizes the human impact of design decisions (12), the role of the physical environment in transactional processes where the relations and interactions between people and the environment are seen as universal conditions of life (11). This whole comprises complexity in several ways—a wide variety of individual capacities that change over time and a diversity of human interactions with the environment (12). Where scientific or medical knowledge is reductionist by nature, a challenge for the relational and holistic methodological framework is to manage the extent of details. A strategy for not “falling into a mind-numbing holism” (29) is to follow the relevant actors. One of the main contributions to the understanding of holism derived from the action research project is the frontloading of the tacit knowledge implicated by the rehabilitation processes and embedded in personal, environmental, and professional spheres or subnetworks. To follow the change processes within BLBL, the individual or personal, the environmental, and the social/professional aspects, were investigated as relational and dynamic characters. Within a small section of the participants' everyday lives, the BLBL's limited scope of lighting + activity + issues allowed an exploration of their interactions and entanglements as a whole constituted by human and non-human actors (28).

**Figure 5:**
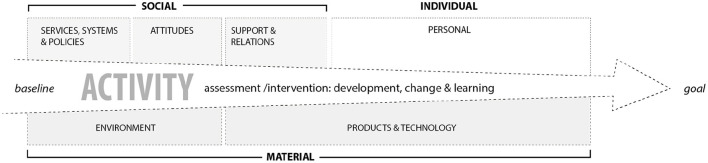
The assessment and intervention with basis in the situated activity work as a trajectory for the change and learning processes across the different parameters, across the social, individual, and material.

The mapping of the parameters in Figures 3, 4 was done with the ICF framework and components in mind. All environmental factors were included in the intervention, however the two intermediate spatial categories introduced in this analysis, building space and illuminated space, encompass the entanglements of environmental factors, but also personal aspects, as motivation and change processes. The activity was the core in both the lighting assessment in the home and the lighting intervention in the lab, and as shown in Figure 5 this core was surrounded by social, individual, and material. Furthermore, the relational mapping incorporates the temporal and processual scope of the GAP model. The findings show that material artifacts are crucial in order to facilitate and support the rehabilitation and recovery processes. The process of identifying, assessing, and weaving together the components and parameters and the relevant actors across the personal and environmental, is embedded in the professional practices. By supporting changes in the home environment or changes in the participants' approach to and use of their environment, the low vision consultant helped enable the abilities of the visually impaired.

However, complexity is difficult to categorize. Identifying the parameter of “social environment” as an environmental and not a personal factor in the framework is a reduction. The field observations showed that the social context, the family or kin, was entangled with both the personal and the professional as an important aspect of how the lighting was enacted as a personal and social routine (34). Another problematic element of classification is how to consider assistive aids such as a pair of glasses, magnifier, or white cane. In many STS studies, assemblages of human and non-human actors are seen as “hybrids,” where an either/or classification would be problematic. In a socio-constructivist framework, all these categories and components are constructed entities that by nature do not encompass hybrids, transitions, or changes.

In other organizational or institutional contexts, new relevant actors will be revealed. When discussing BLBL across the community of low vision consultants, the local management and local political environments highlighted differences. In these contexts, the evidence in the evidence-based practice makes an even larger impact to mobilize moral and economical support for initiating change. In contexts of different professional fields than occupational therapy, the methodological freedom of choice has been a core case of interest. Consequently, the socio-constructivist approach acknowledges the act of balancing the practical expertise of navigation and the structural framework assuring non-discrimination.

### Generalizability

*The definitions used in the ICF have inclusions that provide specifications, synonyms and examples that take into account cultural variation and differences across the life span. It is therefore suitable to be used in different countries and cultures. The ICF can be applied across the entire life span and is suitable for all age-groups (**2**)*.

The very nature of the classification and its codes, one could argue, is to de-contextualize and systematize, and there is consequently a paradox in evidence-based practices, regarding the holistic. One of the challenges lies in the scientific approach: To grasp the role of the environment as part of the participants' everyday lives, we must acknowledge the specific interaction and the very specific environment. The personal and accumulated relationship holds an entanglement of perceptions, values, materialities, and affordances that is completely omitted in a neutralized environment. The importance of a standardized environment was emphasized in relation to the capacity qualifier, which indicated that the environmental factor in this context provides a narrow and mechanical backdrop to a performance or an activity. Similar to the “naked person assessment,” it does not acknowledge the social or personal aspects:

*The Capacity qualifier assumes a “naked person” assessment, that is, the person's capacity without personal assistance or the use of assistive devices. For assessment purposes, this environmental adjustment has to be the same for all persons in all countries to allow for international comparisons. For precision and international comparability, features of the uniform or standard environment can be coded using the Environmental Factors classification (**2**)*.

Assessing interactions and relations within BLBL has enabled an analysis across everyday and professional practices, including the role of the schemes and measures applied in the pilot study. None of the measures related to vision were, in their core structures, related to lighting or the rehabilitation process. The Farnsworth Dichotomous test (D15) and Groffman Visual Tracing Test (GVT) as performance measures represent more scientific and validated schemes developed from the field of ophthalmology and tested over decades. However, they are limited to visual performance and, along with the VFQ-39, these measures represent specific points of time, baselines or endlines, and not the intervening processes. None of the performance measures were specifically developed to assess the impact of lighting or changes in lighting but rather the performance of the participant under the environmental conditions.

The COPM does not directly relate to lighting; however, its focus on activities in combination with the narrative interview and the lighting assessment actively facilitated the process of rehabilitation and recovery. Originally belonging to the fields of psychology, occupational therapy, and lighting theory, these three schemes and methodologies were translated and adjusted to fit the purpose of the pilot study, focusing specifically on the role and use of lighting. Furthermore, they were improved and adjusted within the project as the framework was tested in the individual trajectories of the 60 participants. This can be seen as a classical iterative design process of testing, evaluation, and improvement: How does the framework fit the different needs and processes of the participants and what feels natural to articulate and frame; in what order should the different schemes or questions be introduced? During the conversations with the low vision consultants, it was obvious that, despite the comprehensive design of the COPM and the guidelines for the narrative interview, the application of these schemes differed both due to their application to different types of participants and contexts and the two consultants' different nuances of application. This resembles the complex lived experience of the clients (13, 18–20) and their practices in the recovery process and touches upon issues of rigid schemes not fitting their purposes vs. a lack of structure causing discrimination or incongruities. To acknowledge and accommodate diversity, the framework requires a methodological elasticity or flexibility, while to share and validate practice knowledge, common denominators in schemes, frameworks, and models can help evaluate and make individual practices equitable. In BLBL, a consistent COPM developed quite early in the project, perhaps due to the low vision consultants' existing understanding of the tool from occupational therapy. The test-evaluate-adjust-retest process of the narrative interview and lighting assessment lasted longer, as their role and how they were used and best embedded in the overall process were still being configured throughout the pilot study. As in most innovative processes, closure involves some level of generalization, and the vision consultants found the level of elasticity that worked for them. Subsequently, to transfer their knowledge from the pilot study to their regular practice or across the community of practice, their understanding of the narrative interview and lighting assessment must be specified and translated for the knowledge to be recontextualized (17). A possible next step could be to test these in other contexts to verify their scientific robustness and practical usability.

### Interdisciplinary

In this analysis, a relational model of the physical environment was constructed from the parameters and aspects identified as relevant actors in BLBL, observed or assessed in real life and the associated documents of the 60 cases. The model represents the potential actors (or factors) that could be relevant in future cases but, rather than a checklist of a neutral environment, it works as a framework, a game board, or arena where the collaboration, learning, and change processes occur.

Some scholars accuse the ICF of remaining too closely aligned with a medical understanding of disability and identify universal design (UD) as a more appropriate framework for interdisciplinary collaboration: “Understanding disability from the perspective of the interaction between the individual and the social, cultural and physical environment” (9). Others acknowledge the ICF's support of cross-disciplinary collaboration across different paradigms and individual-social or ideal-material ranges in the field of disability (36). This article was not intended to test either of these but rather to investigate the different frameworks. One of the commonalities is the focus on situated actions in activities, participation, and usability. In one of our conversations on rehabilitation and recovery, the consultants described the ICF as a frame of reference that was embedded in their overall approach and schemes. They had previously used parts of the classification to assess new citizens in their databases; however, in their current practice and BLBL, the ICF was an underlying frame of reference.

One of the ways the ICF is embedded in their practice is through their professional backgrounds as occupational therapists. The complex dynamic relationships between people, occupations, and environments have been a core interest of occupational therapists and the Person-Environment-Occupation (PEO) model was introduced in the 1990s as a practical analytical tool to assist problem analysis, intervention planning and evaluation, or to communicate occupational therapists' practices (37). Similar to the GAP model, PEO holds a processual focus that, in addition to the person–environment, focuses on the occupation on the relational development of the spheres, being more or less congruent within a temporal scope. The PEO model has not been articulated within the framework of BLBL but has been used as an analytical framework for investigating the practice knowledge of low vision consultants in Øien (16), showing that the elasticity of the framework enabled interventions in the more complex settings of the home environment and that it depended on active collaboration between consultants and participants. PEO is distinct from the ICF and UD as a highly practical tool not for classifying or conceptualizing but rather for facilitating evaluation, interventions, and assessments (37).

In their preparations for scaling BLBL to other colleagues across their community of practice, the consultants have been facing the need to generalize and make guidelines for sharing their knowledge, especially their more implicit knowledge. After the pilot BLBL study, the low vision consultants have adjusted the framework for their narrative interview, specifying the ICF components within the questions of the guidelines: *Activity, participation, personal factors, health condition, body function and structure*, and *environmental factors*. By stating the ICF affiliation of each question, the environmental factors were specified in relation to the two main questions: “In what situations do you need to turn the light on?” and “Are there situations where you prefer not to turn the light on?” and the two sub-questions: “When did you last succeed [in performing the specific activity]?” and “What do you think enabled the performance?” The reconfiguration of the interview guide has made the use of the ICF explicit and operationalized it in this specific intervention. The main questions illustrate the relational character of the approach as they include *activity, participation, personal factors*, and *environmental factors*.

Like involving psychologists in the use of the narrative interview to learn the personal aspects, the involvement of an architect and researcher in the field of the built environment has highlighted the role of the physical and material environment and rendered it visible for the low vision consultants. Already part of their tacit knowledge but now articulated and made explicit, they have been able to actively reflect upon its role, enabling a less uniform and neutral understanding of the environmental factors. A shift similar to the understanding of the human body from the medical to the biopsychosocial approach, from a uniform and neutral understanding of the human body to acknowledging the situatedness of lived experiences and human function. As part of the BLBL, the two low vision consultants have acquired both theoretical and hands-on knowledge of lighting, refining the environmental asset in their approach. The analysis explicates this otherwise tacit practice knowledge of acknowledging, recognizing, assessing, relating, and supporting the transformation of the abilities and disabilities of the participants' everyday lives by putting the biopsychosocial approach in motion. Consequently, mapping the interaction as presented in this article enables us to recognize entanglements across the parameters and to identify the relevant human and non-human actors in the rehabilitation processes at stake.

Both in theory and in practice, we address different contexts: the everyday context of the citizen, the professional context of the practitioners, and the political context of systems and legal frameworks. Acknowledging these overlapping contexts would, in Annemarie Mol's understanding of the multiple body (31), allow for collaboration and coordination across practices. Her study was situated in a hospital setting with patients and relatives visiting, while in this study, the professionals visit the home setting. Moreover, where her study investigated several different practices enacting different versions of atherosclerosis, BLBL shows how different versions of lighting can be enacted through one approach. BLBL was originally mobilized bottom-up by practice and informed by ophthalmology, occupational therapy, and psychology but as demonstrated in the action research collaboration, its implementation has been further supported by lighting, architectural anthropology, and socio-material frameworks. Recently, the project group has recognized the potential of refining the participants' knowledge of the learning and change processes by addressing the didactical and collaborative aspects. The methodological and theoretical reflections within our interdisciplinary collaboration have primarily been based on acknowledging these multiple understandings as in the iterative processes within BLBL of recognizing and incorporating the relevant actors in the process, allow us to relate, navigate, and coordinate between the different fields of interest.

When the ICF was launched, the environmental factor classification was highlighted as one of the major innovations within the framework (1), yet today, two decades after its introduction, we argue that operationalizing this factor in practice is pivotal for reducing the gap between the person and the environment to enable disabilities. Several initiatives are working on this issue from different perspectives and in slightly different scopes and we must consider several versions of the ICF as well to use it as classification, as a point of shared reference, and embedded in practical and profession-adjusted tools. We believe that the best way to reduce the gap is by the effort of several forces including the individual and their rehabilitative support and by combining the experience-based tacit knowledge of these with interdisciplinary knowledge of science and technology. One way to approach a common denominator for the human–environment relationship is to acknowledge the physical environment not as a neutral parameter but as a key actor that can enable change.

## Summary and Conclusion

Mapping the role of the physical environment in practice shows that different aspects of it can disable an individual; however, by investigating their abilities via the interrelation and interaction between the individual and the environmental aspects, the relationship can be reintegrated, and abilities regained. By incorporating the participants' experiences in specific activities and specific lighting scenarios and involving embedded and embodied situated knowledge, the low vision consultants supported the process of operationalizing and transforming the person–environment relationship.

The analysis shows that the nature of a model depends on how and where it is used. The physical environment is neutral in randomized controlled trials, standardized in classifications, and yet interdependent and dynamic as part of our living entanglements. In this sense, the context of the classification and the practice of classifying hold different logics and objectives than the context of the rehabilitation process and the way the model informs and operationalizes the practice of facilitating and supporting a change process. In practice, the personal, social, and environmental spheres are not isolated but entangled and, in the rehabilitation process, the professional sphere is also involved. Aspects of the individual of importance to the intervention were their experience of the situation and the lighting scenario, and their motivation. Behavior patterns and experiences are mentioned as personal factors even though this category, due to large societal and cultural variance and lack of clarity are still to be developed in ICF. The level of motivation, which could be argued is particular to the individual, is however classified as a body function, as temperament and personality, which also was persistent in the intervention. Similarly, coping, or managing is discussed in relation to participation, but not classified as a factor as such.

For the contextualized individual, in a specific social and physical environment, the different parameters isolated in factors in the classification, is entangled and interrelational. They are often contained in one another in a way the hierarchical system cannot embrace. The nature of humans and our technologies and environments contains hybrids, where the right adaptions and changes make the individual take on new grounds. The ANT approach enables us to recognize the dynamic and collaborative processes of the intervention as well, where the consultants and the participants identifies, assess, and adapt both the individual and the environmental factors in a joint process.

The understandings and intentions embedded in the ICF, such as the holistic interpretation of health or a focus on functions and abilities, are shared by other scientific and technological approaches such as universal design, that can help us refine our understanding and active involvement of the physical environment. Furthermore, the multiple understanding of the interrelation of people and the environment allows the tacit knowledge of this project to be shared and disseminated not only for low vision rehabilitation and rehabilitation overall but also to the makers of future enabling environments.

Adding new notions of the holistic to the paradigm of rehabilitation includes the dynamic and elastic relationships of spheres in the rehabilitation process and that working across multiple versions enables close collaboration with the participants and interdisciplinary collaboration.

## Data Availability Statement

The original contributions presented in the study are included in the article/supplementary material, further inquiries can be directed to the corresponding author/s.

## Ethics Statement

Ethical review and approval was not required for the study on human participants in accordance with the local legislation and institutional requirements. The patients/participants provided their written informed consent to participate in this study.

## Author Contributions

TØ: concepts, design, data analysis, interpretation, and drafting the article.

## Funding

Velux Foundation have funded the overall research project (Grant Number 00022455) and the work in this article.

## Conflict of Interest

The author declares that the research was conducted in the absence of any commercial or financial relationships that could be construed as a potential conflict of interest.

## Publisher's Note

All claims expressed in this article are solely those of the authors and do not necessarily represent those of their affiliated organizations, or those of the publisher, the editors and the reviewers. Any product that may be evaluated in this article, or claim that may be made by its manufacturer, is not guaranteed or endorsed by the publisher.
